# Cost–utility analysis of longstanding exercise therapy versus usual care in people with rheumatoid arthritis and severe functional limitations

**DOI:** 10.1080/03009742.2024.2392360

**Published:** 2024-10-01

**Authors:** MMH Teuwen, SFE van Weely, CHM van den Ende, MAT van Wissen, TPM Vliet Vlieland, WF Peter, AA den Broeder, D van Schaardenburg, MGJ Gademan, WB van den Hout

**Affiliations:** 1 Department of Orthopaedics, Rehabilitation and Physical Therapy, Leiden University Medical Center, Leiden, The Netherlands; 2 Institute of Allied Health Professions, HU University of Applied Sciences, Utrecht, The Netherlands; 3 Department of Rheumatology, Radboud University Medical Center, Nijmegen, The Netherlands; 4 Department of Research, Sint Maartenskliniek, Nijmegen, The Netherlands; 5 Reade, Center for Rehabilitation and Rheumatology, Amsterdam, The Netherlands; 6 Department of Rheumatology, Sint Maartenskliniek, Nijmegen, The Netherlands; 7 Department of Clinical Epidemiology, Leiden University Medical Center, Leiden, The Netherlands; 8 Department of Biomedical Data Sciences, Leiden University Medical Center, Leiden, The Netherlands

## Abstract

**Objective:**

To evaluate the cost-effectiveness of longstanding personalized exercise therapy compared with usual care in people with rheumatoid arthritis (RA) and severe functional disability.

**Method:**

In this cost–utility analysis of a randomized controlled trial (n = 215), with 1 year follow-up, the study population comprised individuals with RA and reported severe difficulties in performing basic daily activities. Assessments were at baseline, 12, 26, and 52 weeks, with measurements of costs including medical and non-medical costs as recorded by patients and healthcare providers. Quality-adjusted life-years (QALYs) were estimated using the EuroQol 5 dimensions 5 levels (EQ-5D-5L) and EuroQol Visual Analogue Scale (EQ-VAS). Costs and QALY differences were analysed according to the intention-to-treat principle using cost-effectiveness acceptability curves.

**Results:**

The 1 year societal costs were non-significantly in favour of the usual care group, with a small difference of €180 [95% confidence interval (CI) €−4493 to €4852]. The QALYs were non-significantly in favour of the intervention group, by 0.02 according to the EQ-5D-5L (95% CI −0.05 to 0.09) and by 0.04 according to the EQ-VAS (95% CI 0.00 to 0.08). For a willingness-to-pay threshold of €50 000 per QALY, the intervention was the cost-effective strategy with 60% certainty.

**Conclusion:**

This economic evaluation showed no clear economic preference for either group, as the intervention costs were higher in the intervention group, but partly compensated by other cost savings and improved QALYs. Despite severe RA, patients had better clinical outcomes compared with usual care, suggesting no economic reasons to refrain from exercise therapy.

**Trial Registration number:**

Netherlands Trial Register NL8235, included in the International Clinical Trial Registry Platform (ICTRP) (https://trialsearch.who.int/Trial2.aspx?TrialID=NL8235).

Rheumatoid arthritis (RA) is a chronic autoimmune disease with a substantial impact on the quality of life of individuals (1–3). Current management treatment strategies include pharmacological and non-pharmacological treatments, with exercise therapy being one of the key elements (4–6). Various studies have demonstrated positive effects of supervised exercise therapy on aerobic capacity, muscle strength, functional ability, overall quality of life, and the cardiovascular risk of people with RA (7–13). Based on this evidence, exercise therapy is advocated in various professional guidelines on the management of RA (4–6). A recent randomized controlled trial (RCT) evaluated the effectiveness of long-term exercise therapy with usual care in RA patients with severe functional disability due to complex health problems (14, 15). This study found that a personalized, active 52 week exercise programme comprising education, exercises, and promotion of physical activity was more effective than usual care, with a statistically significant effect on both measures of physical functioning and quality of life (14).

In addition to confirming the effectiveness of exercise therapy interventions, it is important to assess the cost-effectiveness. Integrating economic criteria helps to evaluate whether the additional health gains justify the extra healthcare resources needed. Studies have thus far provided limited information on the cost-effectiveness or cost–utility of exercise interventions in RA, as confirmed in a systematic review and meta-analysis (7). Whereas several economic analyses of exercise therapy in RA were concerned with studies that did not compare a comprehensive exercise therapy intervention with usual care (16–19), one was conducted alongside a 104 week RCT comparing a high-intensity exercise programme with usual care in people with RA (20). This study found higher medical costs and societal costs, without improvement of quality-adjusted life-years (QALYs), and was therefore deemed not cost-effective (20). However, that study did not include patients with a specific indication for exercise therapy, it had a fixed delivery of two sessions per week, and the study took place over 20 years ago, hampering its generalizability to more recent clinical trials in RA patients with specific health problems.

A more relevant economic analysis in the context of the RCT in people with RA and complex functional problems (14) is an RCT on goal-oriented exercise therapy for frail older adults with mobility limitations (21, 22). That study compared the (cost-)effectiveness of a patient-centred physical therapy strategy (Coach2Move) with standard treatment, showing significant cost savings and improved QALYs (21). Despite its promising results, this study was conducted in a population of frail elderly people with mobility problems, which may not be generalizable to people with RA and functional disability.

Given the lack of knowledge on the cost-effectiveness of exercise therapy in people with RA and severe functional limitations, the objective of the present study was to evaluate the cost-effectiveness of longstanding, personalized exercise therapy compared with usual care in people with RA and severe functional limitations.

## Method

### Study design

We conducted an economic evaluation alongside a 52 week RCT (the L-EXTRA study) comparing personalized exercise therapy with usual care (14, 15). The sample size of 215 patients was determined based on the primary outcome measure of the L-EXTRA study, i.e. the patient specific complaints. More information on the design of the study can be found in the published study protocol (15). The cost–utility analysis, adhering to Dutch economic evaluation guidelines (23, 24), was coordinated by Leiden University Medical Center between October 2019 and June 2023. The study was approved by the Medical Ethical Review Board Leiden–Den Haag–Delft (METC-LDD, P19.052), registered in the Dutch Trial Register (ClinicalTrials.gov: NL8235), and all patients provided written informed consent.

### Study population

The study population comprised people with RA and severe functional disability, defined as self-perceived difficulties in basic daily activities (for eligibility criteria, see Table 1). The definition of functional disability had been established by an expert group consisting of patients, rheumatologists, healthcare professionals, and researchers. In people with RA, functional disability has a well-established link with disease activity (25–27), but it can also be due to other factors such as joint damage or comorbidities (25, 27). In part, the population of people with RA and severe functional disability may overlap with those meeting the criteria of difficult-to-treat RA (5, 28, 29).

**Table 1.** Eligibility criteria for participation in the L-EXTRA (Longstanding EXercise Therapy in patients with Rheumatoid Arthritis and severe functional disabilities) study.

Inclusion criteria
1. Adults (aged ≥ 18 years) with a rheumatoid arthritis diagnosis confirmed by a rheumatologist
2. Individuals who experienced severe limitations in basic daily activities, including self-care (e.g. dressing, washing), making transfers (e.g. getting into and out of bed, rising from a chair, using the toilet), and/or mobility indoors or outdoors
3. Individuals whose limitations were directly or indirectly associated with their rheumatic condition, e.g. with persisting or progressive disease activity despite optimal pharmacological treatment, severe joint damage or deformities, and/or severe comorbidities (e.g. pulmonary or cardiovascular disease, depression, obesity)
4. Individuals whose functional limitations were unlikely to be improved by or resolved with a short exercise therapy intervention
Exclusion criteria
1. Individuals who had been individually treated by a physical therapist currently or in the past 3 months, whether or not in the context of a multidisciplinary team intervention
2. Individuals who needed imminent admission to a hospital or rehabilitation centre
3. Individuals who were unable to provide informed consent

### Recruitment and selection procedures

During a 22 month recruitment period, study information was disseminated via various channels. To reach potential participants, information was shared through websites, digital newsletters, flyers, and posters. Rheumatologists and clinical nurse specialists received information via e-mails and presentations. In addition, information letters were mailed to potentially eligible RA patients at two centres (Sint Maartenskliniek, Nijmegen; and Reade, Amsterdam).

Patients with RA who were interested could register themselves or via their treating clinician. The screening for eligibility consisted of a telephone interview with one researcher and subsequent discussion with two other research team members. If they were unsure about a patient’s eligibility, the larger research team was consulted and/or the patient and/or the treating rheumatologist were contacted. For those patients fulfilling the eligibility criteria, the treating rheumatologist was asked to confirm the clinical diagnosis of RA.

### Intervention and control conditions

The intervention consisted of personalized, supervised, longstanding (≥ 52 weeks) active exercise therapy. The intervention was delivered by trained primary care physical therapists working in the neighbourhood of the participants, either at the practice of the physical therapist or in the participant’s home. The mandatory training for physical therapists was provided via an online education session or app. The intervention was provided according to a standardized treatment protocol, including initial assessment, setting of treatment goals, and provision of active treatment, with individual adjustments based on regular monitoring and evaluations. More details of the intervention protocol have been published previously (14, 15). Participants randomized to the control group received usual care, to be determined by the treating clinician(s) and the patients. Usual care could include usual physical therapy, by referral or self-referral, but only if provided by a physical therapist who did not treat participants in the intervention group. After 52 weeks, participants in both the intervention and usual care groups had access to the intervention until the end of the study.

### Assessments

#### Sociodemographic and disease characteristics

After enrolment, sociodemographic and health characteristics were collected from the patients using a questionnaire comprising questions on age (years); sex (male/female/other); height (cm) and weight (kg), to calculate the body mass index (BMI); single person household (yes/no); educational level [low/medium: primary or secondary (vocational) education; high: bachelor’s or master’s degree at a university (of applied sciences)]; if 66 years or younger, having a paid job (yes/no); additional health insurance coverage (yes/no); self-reported duration of complaints (years); Health Assessment Questionnaire Disability Index (HAQ-DI) score (0–3) (30); smoking status, currently or ever smoked (yes/no); and presence of 19 different comorbidities (yes/no), based on a questionnaire developed by Statistics Netherlands (31). In addition, the treating rheumatologist was asked to provide information on the fulfilment of the definition of difficult-to-treat RA (yes/no) (5, 29) and the years since diagnosis (years).

#### Utility measures and QALYs

Utility reflects the value of quality of life, on a scale anchored at 0 (‘as bad as death’) and 1 (‘perfect health’). We measured utility in two ways, at baseline, and at 12, 26, and 52 weeks. Participants described their general health status using the EuroQol 5 dimension 5 levels (EQ-5D-5L) classification system (32). From the EQ-5D-5L classification system, the Dutch utility index was calculated (33). Also, patients rated their health status using the EuroQol Visual Analogue Scale (EQ-VAS), ranging from 0 to 100, where 0 indicates the worst imaginable health and 100 the best imaginable health (32, 33). The obtained EQ-VAS values were transformed into a utility score using the power function 1 − (1 − (EQ-VAS/100))^1.61^ (34). One-year QALYs are a commonly used measure to inform healthcare resource allocation decisions (35), and were calculated by the area under the curve of each of the utility measures over the follow-up period.

#### Costs

One-year societal costs were calculated at the 2023 price level. Participants filled out questionnaires at 12, 26, and 52 weeks on healthcare use, domestic help and informal care, and hours of paid working time, absenteeism, and presenteeism, and lost unpaid productivity [adapted from the institute for Medical Technology Assessment (iMTA) Medical Consumption Questionnaire and the iMTA Productivity Cost Questionnaire] (36, 37). In the intervention group, physical therapists reported the number of sessions for each study participant. In addition, participants in the intervention group were asked about the number of sessions of physical therapy. When participants reported more physical therapy sessions than their physical therapist had registered, the difference was designated as the discrepancy between patient-reported and physical therapist-registered physical therapy. All physical therapy sessions reported by participants in the usual care group were counted as ‘patient-reported physical therapy’. Where possible, healthcare was valued with Dutch standard prices (38, 39); otherwise, market prices were used. Travel costs were calculated from the number of healthcare visits, combined with national averages on travel distance and means of transportation (24). Domestic help, informal care, and unpaid productivity were all valued at €17 per hour (24). According to the friction method, paid productivity losses were valued at €42 per hour, with a maximum of 3 months productivity loss (24, 38, 39).

### Statistical analysis

Data were analysed using IBM SPSS for Windows, version 25.0 (released 2017; IBM Corp., Armonk, NY, USA). All statistical comparisons were performed using standard unequal variance t-tests according to the intention-to-treat principle, meaning that data from participants were analysed according to the randomized treatment assignment. Multiple imputation with 100 imputed data sets was used to account for missing data and to preserve power and possibly reduce bias (40). Cost-effectiveness acceptability curves were constructed to show the probability that the longstanding exercise therapy was cost-effective compared with usual care, depending on the societal willingness to pay (WTP) for an additional QALY. These curves were calculated as the one-sided p-value for the differences in Net Benefit = (WTP × QALY) – Costs, between the patients in the intervention and the usual care groups (41). In the Netherlands, acceptable WTP levels range from €20 000 to €50 000 or €80 000 per QALY (42), with €50 000 per QALY considered most suitable for the current patient population. In the base-case economic evaluation, total societal costs were compared to QALYs based on the utility index calculated from the EQ-5D-5L. Incremental cost-effectiveness ratios were calculated as the difference in costs divided by the difference in QALYs, but were not further formally analysed. Sensitivity analyses were performed by considering a different utility measure (EQ-VAS) and different cost measures (costs from a medical perspective and only intervention costs).

## Results

In total, 215 participants were included in the study: 109 participants in the intervention group and 106 in the usual care group. The mean ± sd ages of participants were 59.4 ± 12.1 and 58.1 ± 13.6 years, the proportions of females were 89% and 92%, and the proportions of patients with one or more joint arthroplasties were 38% and 37% in the intervention and usual care groups, respectively. There were no relevant differences in baseline characteristics between the groups (Table 2). Over the course of the 1 year study, 11 participants discontinued their participation (14). In terms of health resource utilization and productivity measurements, 7% of the data was missing, and for utility measurements, 5% of the data were missing.

**Table 2.** Baseline demographic and health characteristics of people with rheumatoid arthritis (RA) and severe functional limitations participating in a randomized controlled trial comparing the cost–utility of longstanding personalized exercise therapy with usual care.

	Intervention group (N = 109)	Usual care group (N = 106)
Female	97 (89.0)	97 (91.5)
Age (years)	59.4 ± 12.1	58.1 ± 13.6
BMI (kg/m^2^)	27.2 ± 5.0	27.9 ± 6.9
Single-person household	30 (27.5)	37 (34.9)
Higher education*	35 (32.1)	27 (25.5)
Work status		
≤ 66 years old	82 (75.2)	72 (67.9)
Paid job	23 (28.0)	22 (30.6)
No job, health problems	32 (39.0)	29 (40.3)
No job, other reasons	27 (32.9)	21 (29.2)
Health insurance with additional coverage	96 (88.1)	98 (92.5)
Self-reported duration of complaints (years)	21.6 ± 12.6	21.6 ± 14.0
Years since diagnosis (years)	18.0 ± 11.9 (N = 102)	19.7 ± 14.1 (N = 91)
Difficult-to-treat RA (EULAR criteria)^†^	44 (43.6) (N = 101)	46 (51.1) (N = 90)
HAQ-DI	1.7 ± 0.5	1.7 ± 0.5
Smoking status: ever smoked	60 (55.0)	68 (64.2)
Number of comorbidities	(N = 108)	(N = 105)
0	3 (2.8)	5 (4.8)
1–2	23 (21.3)	28 (26.7)
3–4	39 (36.1)	33 (31.4)
≥ 5	43 (39.8)	39 (37.1)

Data are shown as n (%) or mean ± sd.

* Higher education: Bachelor’s or Master’s degree at university (of applied sciences).

^†^Definition of difficult-to-treat RA based on Nagy et al (29).

BMI, body mass index; EULAR, European Alliance of Associations for Rheumatology; HAQ-DI, Health Assessment Questionnaire Disability Index.

### Utilities and clinical outcome

**Figure 1.**
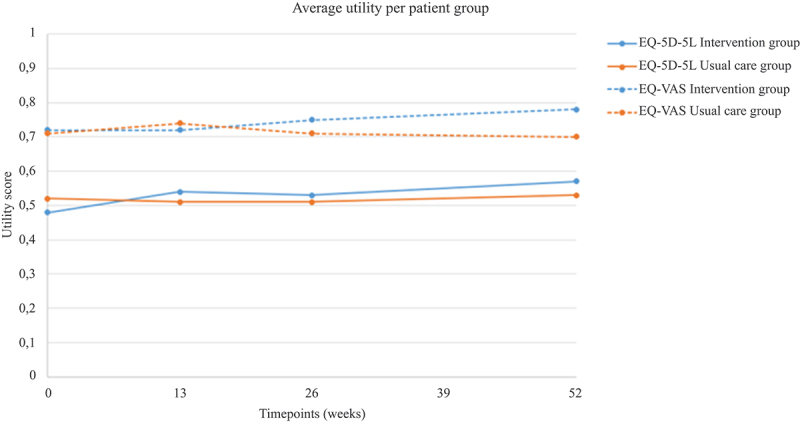
Mean utility, by time and treatment group. EQ-5D-5L, EuroQol 5 dimensions 5 levels; EQ-VAS, EuroQol Visual Analogue Scale.

Table 3 reports the EQ-5D-5L and EQ-VAS at each time-point. The mean valuation of health for both utility measures after 1 year was more favourable in the intervention group than in the usual care group (Figure 1). However, with the exception of the difference in the EQ-VAS score at 52 weeks [0.09 point, 95% confidence interval (CI) 0.03 to 0.14, p = 0.001], none of the differences reached statistical significance.

**Table 3.** Mean utility scores and quality-adjusted life years (QALYs), by time and group.

	Intervention group (N = 109)	Usual care group (N = 106)	MD*	95% CI	p^†^
EQ-5D-5L utility score					
Baseline	0.48 ± 0.28	0.52 ± 0.26	−0.04	−0.11 to 0.03	0.31
12 weeks	0.54 ± 0.28	0.51 ± 0.28	0.03	−0.04 to 0.11	0.42
26 weeks	0.53 ± 0.32	0.51 ± 0.27	0.02	−0.06 to 0.10	0.66
52 weeks	0.57 ± 0.29	0.53 ± 0.26	0.04	−0.03 to 0.12	0.28
QALY EQ-5D-5L	0.54 ± 0.25	0.52 ± 0.22	0.02	−0.05 to 0.09	0.54
EQ-VAS utility score					
Baseline	0.72 ± 0.19	0.71 ± 0.19	0.01	−0.04 to 0.06	0.66
12 weeks	0.72 ± 0.18	0.74 ± 0.19	−0.02	−0.07 to 0.03	0.43
26 weeks	0.75 ± 0.19	0.71 ± 0.19	0.05	−0.01 to 0.10	0.09
52 weeks	0.78 ± 0.16	0.70 ± 0.22	0.09	0.03 to 0.14	0.001
QALY EQ-VAS	0.75 ± 0.14	0.71 ± 0.16	0.04	0.00 to 0.08	0.08

Data are shown as mean ± sd. Mean values are shown for each assessment point (baseline, and 12, 26, and 52 weeks).

*Mean differences in QALYs between the intervention and usual care groups.

^†^The p-values and 95% CIs refer to the differences in QALYs between groups.

MD, mean difference; CI, confidence interval; EQ-5D-5L, EuroQol 5 dimensions 5 level; EQ-VAS, EuroQol Visual Analogue Scale with power transformation.

### Cost of exercise therapy

Table 4 shows the costs per patient in the intervention and usual care groups. The proportion of participants in the intervention group who had actually used the longstanding personalized exercise therapy intervention was 91%. The mean number of sessions based on the registrations of the physical therapists was 35 for the total group and 39 for the group who had used the intervention, with the large majority of treatments provided at the physical therapist’s practice. The mean total costs of the intervention in the intervention group were estimated at €1423 ± 762 per patient. Compared to the registrations of the treating physical therapists, 46% of the participants in the intervention group reported more treatment sessions than the number registered by the physical therapist. This discrepancy could be due to underreporting by physical therapists, overreporting by patients, or the use of physical therapy outside the study intervention, although the last of these was discouraged. The mean costs of physical therapy that was reported by patients but not registered by physical therapists in the intervention group were €433 ± 722.

**Table 4.** Mean 1 year medical and non-medical costs per participant, by group.

	Intervention group (N = 109)		Usual care group (N = 106)		Difference in costs between groups		
	Volume	Costs (€)	Volume	Costs (€)	Difference (€)	95% CI	p
Physical therapy (visits)							
Intervention, at centre*	87% 39	1361	2% 19	14	1346	1194 to 1499	< 0.001
Intervention, at home*	2% 41	32	0% –	0	32	−14 to 78	0.17
Intervention, combination	2% 41	30	0% –	0	30	−12 to 72	0.16
Total intervention physical therapy* (mean ± sd)	91% 39	1423 ± 762	2% 19	14 ± 127	1409	1264 to 1554	< 0.001
Patient-reported physiotherapy*			66% 24	644	−211	−431 to 9	0.06
Discrepancy in patient-reported and physical therapist-registered physiotherapy*	46% 23	433					
Total physical therapy (mean ± sd)	98% 47	1856 ± 815	68% 24	659 ± 851	1198	967 to 1428	< 0.001
General practitioner	5.5	194	6.4	222	−28	−79 to 24	0.29
Specialists							
Rheumatologist (visits)	4.3	477	4.2	467	10	−73 to 93	0.81
Orthopaedic surgeon (visits)	0.9	104	1.0	114	−10	−57 to 38	0.69
Internist (visits)	0.7	77	0.4	39	37	0 to 75	0.05
Cardiologist (visits)	0.6	65	0.4	49	16	−20 to 51	0.39
Other (visits)^†^	3.0	336	2.1	235	101	−47 to 249	0.18
Other healthcare providers							
Rheumatology nurse (visits)	1.9	40	1.3	27	13	−6 to 32	0.18
Podiatrist (visits)	0.8	31	0.6	25	6	−11 to 23	0.50
Occupational therapist (visits)	0.9	34	1.0	40	−5	−47 to 34	0.78
Dietitian (visits)	1.0	36	0.6	22	14	−12 to 41	0.29
Social worker (visits)	0.3	27	0.4	32	−5	−41 to 32	0.80
Other (visits)^‡^	1.3	93	1.4	57	36	−8 to 81	0.41
Day treatment hospitalizations							
Hospital (visits)	1.5	448	1.1	343	106	−140 to 351	0.40
Rehabilitation centre (visits)	0.1	84	1.3	817	−733	−1926 to 461	0.23
Psychotherapeutic institution (visits)	1.5	350	2.1	490	−140	−636 to 357	0.58
Inpatient hospitalization							
Hospital (days)	2.3	1341	2.0	1168	173	−1107 to 1453	0.79
Rehabilitation centre (days)	1.4	788	2.2	1255	−467	−1728 to 794	0.47
Psychotherapeutic institution (days)	0	0.0	0	0.0	–	–	–
Home care (h/week)	0.7	1575	0.7	1612	−37	−854 to 779	0.93
Medication							
bDMARDs	79%	6187	79%	5348	839	−678 to 2356	0.26
tsDMARDs	40%	2252	51%	2693	−441	−2626 to 1744	0.69
csDMARDs	81%	220	65%	170	50	−21 to 121	0.17
NSAIDs	65%	162	59%	146	16	−60 to 92	0.68
Corticosteroids	72%	14	65%	11	4	−1 to 9	0.13
Total medication costs (mean ± sd)	100%	8834 ± 8367	98%	8367 ± 8001	467	−2329 to 3264	0.74
Total medical costs (mean ± sd)		16 791 ± 11 361		16 038 ± 12 454	754	−3012 to 4519	0.70
Non-medical costs							
Working hours (h/week)	5.5		5.1				
Absenteeism (h/week)	1.3		0.7				
Presenteeism (h/week)	1.4		1.9				
Productivity cost	–	2447	–	2026	421	−1014 to 1856	0.57
Lost unpaid labour (h/week)	2.8	2446	3.3	2949	−503	−1909 to 904	0.48
Household help (h/week)	0.4	394	0.7	590	−196	−528 to 137	0.25
Informal care (h/week)	4.7	4157	5.1	4521	−364	−1786 to 1058	0.62
Travel costs physiotherapy		101		37	65	52 to 78	< 0.001
Travel costs other healthcare		104		101	2	−22 to 26	0.84
Total non-medical costs (mean ± sd)		9650 ± 9568		10 224 ± 8788	−574	−3098 to 1950	0.66
Total societal costs (mean ± sd)		26 441 ± 16 021		26 261 ± 15 321	180	−4493 to 4852	0.94

* The number of visits reported here is the average among patients with at least one visit.

^†^Other healthcare specialists, i.e. surgeon, gynaecologist, pulmonologist, dermatologist, neurologist, ophthalmologist, or urologist.

^‡^Other healthcare providers, i.e. psychologist, nurse other than rheumatology specialist nurse, medical pedicure, speech therapist, skin therapist, or practice assistant.

bDMARD, biological disease-modifying anti-rheumatic drug; CI, confidence interval; csDMARD, conventional synthetic disease-modifying anti-rheumatic drug; NSAID, non-steroidal anti-inflammatory drug; tsDMARD, targeted synthetic disease-modifying anti-rheumatic drug.

In the usual care group, 66% of the participants reported the use of physical therapy other than the study intervention, with the mean costs per patient being €644 ± 852. Owing to a logistical error, two patients (2%) in the usual care group were also given access to the intervention within the 52 week period, resulting in mean costs of the intervention in the usual care group of €14 ± 127.

### Other medical and non-medical costs

The difference between the intervention and usual care groups in total 1 year medical costs was estimated to be €754 (95% CI €−3012 to €4519), non-statistically significantly in favour of the usual care group (Table 4). This difference was almost exclusively driven by lower inpatient and day-patient rehabilitation costs in the intervention group than in the usual care group, although these were not significantly different. Regarding the costs of medication, the estimated costs in both groups were the highest for biological and targeted synthetic disease-modifying anti-rheumatic drugs (bDMARDs and tsDMARDs), with overall relatively similar proportions of patients using specific types of DMARDs. In general, there were no differences in the estimated costs of medication between the intervention and usual care groups.

With respect to the 1 year non-medical costs, the difference between the intervention and usual care groups was estimated to be €−574 (95% CI €−3098 to €1950), non-statistically significantly in favour of the intervention group. Most of the non-medical costs were attributable to informal care, where more informal care costs were seen in the usual care group than in the intervention group; however, the difference was not statistically significant. There were also more costs in the usual care group for home help and lost unpaid labour, but the differences did not reach statistical significance. The mean total societal costs per participant were estimated at €26 441 for the intervention group and €26 261 for the usual care group, with a difference in total societal costs of €180 (95% CI €−4493 to €4852) in favour of the usual care group. However, this overall difference was not statistically significant.

### Cost–utility analysis

**Figure 2.**
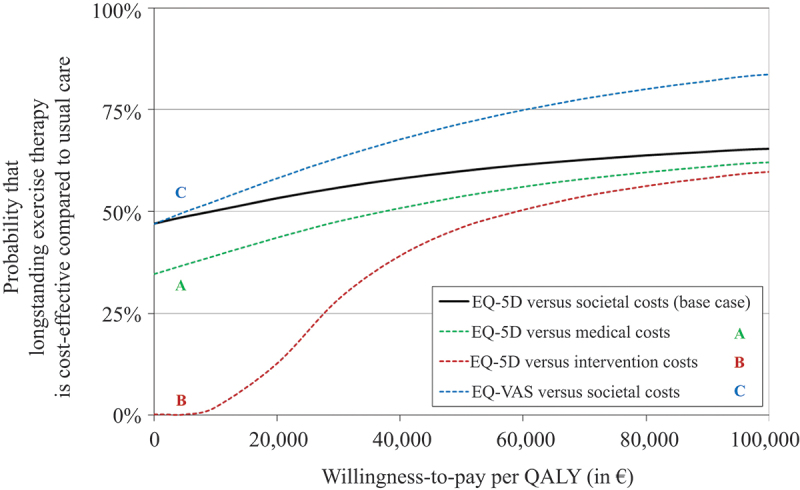
Cost-effectiveness acceptability curve showing the probability that longstanding personalized, supervised exercise therapy compared with the usual care programme is a cost-effective strategy over a range of values for the willingness to pay for an additional quality-adjusted life year (QALY). EQ-5D-5L, EuroQol 5 dimensions 5 level; EQ-VAS, EuroQol Visual Analogue Scale.

In the base-case cost–utility analysis, the total societal costs were somewhat more favourable in the usual care group, whereas QALYs based on the EQ-5D-5L were somewhat more favourable in the intervention group, although both differences were not statistically significant. As a result, the probability that the intervention is cost-effective is below 50% if QALYs are valued low (left-hand part of the acceptability curve in Figure 2) and is above 50% if QALYs are valued high (right-hand part in Figure 2). But, regardless of the WTP, this probability of cost-effectiveness remains close to 50%, ranging from 47% to 65%. For a WTP threshold of €50 000 per QALY, the intervention is 60% likely to be the cost-effective strategy. The point estimate for the incremental cost-effectiveness ratio is favourable at €9000 per QALY; however, this is a very unstable estimate with an infinite confidence interval, owing to the non-significant difference in QALYs. In the sensitivity analyses with QALYs calculated from the EQ-VAS, results were more in favour of the intervention group compared to QALYs calculated from the EQ-5D-5L, with the acceptability curves showing a higher probability for the intervention to be cost-effective, especially for the higher WTP values. Alternatively, the narrower cost perspectives with only healthcare costs or only intervention costs resulted in larger and more statistically significant cost differences in favour of usual care. As a result, the acceptability curves show a lower probability for the intervention to be cost-effective, especially for the lower WTP values. At €50 000 per QALY, the intervention is 46–72% likely to be cost-effective. These results indicate that there is no clear economic preference for either the intervention or usual care: regardless of society’s WTP for an additional QALY, both were about equally likely to be cost-effective.

## Discussion

This economic analysis alongside a 52 week, assessor-blinded RCT, comparing the cost-effectiveness of longstanding personalized exercise therapy with usual care in people with RA and severe functional limitations, concluded with no clear economic preference for either the intervention or usual care. The total intervention costs were €1423, whereas the estimated total societal costs were €180 higher in the intervention than in the usual care group. This difference was small and not statistically significant, with a wide confidence interval owing to the high variability in costs (95% CI €−4493 to €4852). The intervention group had more favourable health valuations than the usual care group, although the differences were not statistically significant, except for the EQ-VAS at 52 weeks. The net-benefit analysis showed that there was no clear economic preference for either group. Regarding the existing literature, economic analyses of exercise therapy in RA have primarily focused on very specific exercise interventions: hand exercise (16), promotion of physical activity only (17), a comparison across two exercise interventions (18), or combinations of exercise with other treatment modalities (19). Thus, a conclusion on the cost-effectiveness of comprehensive exercise therapy interventions, as advocated in professional guidelines on the management of RA (4–6), cannot be drawn. Only one other economic analysis on a somewhat similar intervention to that used in the present study, being a 104 week exercise therapy intervention comprising high-intensity aerobic and muscle-strengthening exercises, has been conducted in patients with RA. From that study, it was concluded that compared with usual care, the intervention provided insufficient improvement in the valuation of health to justify its extra costs (20). An important difference from the present study is that the previous study included a different group of RA patients, not selected based on functional disability, with stable medication, and lacking weight-bearing joint prostheses or serious comorbidities (20). Indeed, the utility scores of the patients included in the previous study were more favourable at baseline than those of the patients in the present study, potentially leading to smaller room for improvement. Moreover, the intervention in the previous study was different, as it concerned a group exercise therapy programme with a relatively fixed content and number of sessions for every patient, whereas the intervention in the current study was explicitly tailored to patients’ individual needs. For these reasons, fair comparisons with the present study are difficult to make.

However, the economic analysis of an intervention that was used in the development of the exercise therapy intervention in the present study is of interest (21). That study was conducted in another population, namely, elderly people with complex mobility problems, yet concerned an intervention tailored to individual patients’ personal functional goals (21, 22). Compared to usual care, the Coach2Move intervention yielded not only cost savings but also an effect on QALYs. Notably, in that study, all patients in the usual care group used physical therapy, whereas in our study the use of physical therapy was left to the discretion of the patient and/or clinicians. Thereby, the costs of physical therapy in the usual care group in our study were much lower than those in the Coach2Move study, making it more difficult to demonstrate the cost-effectiveness of the intervention.

The net costs in our study were slightly higher in the intervention group, primarily driven by the costs of the intervention. Although it could be hypothesized that this intervention may alleviate the necessity for expensive treatments, such as hospital or rehabilitation centre admissions, we were unable to establish any statistically significant difference in this aspect. In addition, while it is plausible that the intervention could improve work ability through enhanced functional capacity, we found no statistically significant difference in this aspect. Here, it must be noted that whereas 75% of the patients were of working age, only 30% of these had a paid job at enrolment. However, an ongoing study is evaluating a physical therapy intervention specifically aimed at working individuals with inflammatory arthritis, including RA (43).

Strengths of the current study include a low attrition rate and minimal missing values on utilities and cost-related assessments, enhancing the generalizability of the study results. In addition, the positive effects with respect to QALYs are in line with the proven benefits according to various self-reported and performance-based health outcomes in a separate analysis (14). A limitation of our study is the 1 year time horizon; and the long-term effects in the context of continued use of exercise therapy need to be further investigated. Another limitation concerns the estimation of the costs of the intervention. There appeared to be a discrepancy between the number of sessions reported by patients and that registered by the physical therapists, possibly owing to overreporting by the patients (including the use of physical therapy other than the intervention), underreporting by physical therapists, or their combination. As we have no other means to verify the true number, the observed discrepancy cannot be resolved. In addition, the study focused on individuals with severe functional limitations, with a mean disease duration of approximately 20 years, most of whom had low employment rates, potentially limiting the scope for improvement. However, results for individuals with milder functional limitations, who may require shorter and intermittent treatment, could be different. Another limitation relates to the generalizability of the results to healthcare settings other than the Netherlands, where access to physical therapy could be different, owing to variations in healthcare and insurance systems.

## Conclusion

The results of this study showed no clear economic preference for either the intervention or usual care: the intervention costs were compensated by other cost savings and improved QALYs. However, underlining that the results apply to patients with longstanding, severe disease, despite which they experienced better clinical outcomes compared with usual care, the results of this study suggest that there are no clear economic reasons to refrain from longstanding exercise therapy in people with RA and severe functional limitations. Considering the severity and possible fluctuation of functional limitations over time, it is to be advised that patients should have permanent access to this intervention, with the actual usage tailored to their current needs.

## Data Availability

The data underlying this article will be shared upon reasonable request to the corresponding author.
